# Comparative Clinical Analysis of Gastroenteropancreatic Neuroendocrine Carcinomas with Liver Metastasis and Primary Hepatic Neuroendocrine Carcinomas

**DOI:** 10.1155/2018/9191639

**Published:** 2018-10-17

**Authors:** Meng-jun Qiu, Yao-bing Chen, Ning-rui Bi, Sheng-li Yang, Xiao-xiao He, Zhi-fan Xiong

**Affiliations:** ^1^Division of Gastroenterology, Liyuan Hospital, Tongji Medical College, Huazhong University of Science and Technology, Wuhan 430077, China; ^2^Institute of Pathology, Tongji Hospital, Tongji Medical College, Huazhong University of Science and Technology, Wuhan 430022, China; ^3^Department of Hepatobiliary Surgery, The Affiliated Hospital of Guizhou Medical University, Guiyang, Guizhou 550001, China; ^4^Cancer Center, Union Hospital, Tongji Medical College, Huazhong University of Science and Technology, Wuhan 430022, China

## Abstract

**Purpose:**

The objective of this study was to analyze the clinical features and prognosis of gastroenteropancreatic (GEP) neuroendocrine carcinomas (NECs) with liver metastasis and primary hepatic neuroendocrine carcinomas (PHNECs), as these rare hepatic neuroendocrine carcinomas have not been exhaustively studied.

**Methods:**

The clinical data of 47 patients with hepatic NECs were retrospectively reviewed and categorized to analyze features and prognosis.

**Results:**

The 47 studied cases comprised 13 cases of primary hepatic NECs (primary group) and 34 cases of metastatic hepatic NECs (metastatic group). Male patients were slightly dominant in both groups, while no age predilection was present. PHNECs were mostly single nodules located in the right lobe of the liver. Metastatic hepatic NECs originated mostly from the pancreas and stomach without distinction of the lobes of the liver. Univariate analysis showed that the treatment protocol (radical operation or others) was correlated with the overall survival (OS; *p* < 0.05) in the primary group, while treatment protocol and cytokeratin 7 (CK7) were associated with OS (*p* < 0.05) in the metastatic group. Cox proportional hazard regression showed that radical operation was an independent prognostic factor (*p* < 0.05) for OS in the metastatic group.

**Conclusions:**

No significant differences in the clinicopathological features between PHNECs and metastatic hepatic GEP NECs were found, but radical operation was significantly correlated with OS for both carcinomas. Radical operation is the first choice for patients who are eligible for operation.

## 1. Introduction

Neuroendocrine tumors (NETs), also known as amine precursor uptake decarboxylation (APUD) tumors, are an uncommon type of cancer originating from disseminated neuroendocrine cells. According to the World Health Organization (WHO), gastroenteropancreatic NETs are categorized into three grades G1 to G3 based on the mitotic rate and the Ki67 index (G1: <2 mitoses/10 high power field (HPF) and Ki67 index <3%; G2: 2–20 mitoses/10 HPF or Ki67 index 3–20%; and G3: >20 mitoses/10 HPF or Ki67 index >20%). NETs of the G1/G2 grade were regarded as well as differentiated. High-grade (G3) neoplasms have been regarded as synonymous with poorly differentiated NECs [1]. Outcome and treatment of NETs and NECs are strikingly different. Extrapulmonary NECs are most often found in the gastrointestinal (GI) tract [2]. The liver is the common site for metastasis, yet it is an uncommon site for the origin of carcinomas [3]. Little is known about PHNECs, and the diagnosis of PHNECs is a problem worthy of discussion. The differential diagnosis between PHNECs and metastatic hepatic GEP NECs is very important for the diagnosis of PHNECs. Due to the rarity and similarity of PHNECs and metastatic hepatic GEP NECs, their clinical features and treatment outcomes are not well understood. In this study, we retrospectively reviewed experiences with these two carcinomas for contributing to the overall understanding and improved distinction of PHNECs and metastatic hepatic GEP NECs.

## 2. Methods

### 2.1. Collection of Clinical Data

Seventy-four patients, who were surgically resected or confirmed by pathological biopsy in Union Hospital and Tongji Hospital, Tongji Medical College, Huazhong University of Science and Technology, between June 2012 and June 2017, were retrospectively reviewed. According to the European Neuroendocrine Tumor Society (ENETS), all patients were classified based on their mitotic figures and (or) Ki67-positive indices. Among all reviewed patients, 47 patients exhibited complete pathological and follow-up data and were thus eligible for histopathological and prognostic analysis. Ethical approval was requested and obtained from the Medical Ethics Committee of Tongji Medical College (Wuhan, China). Written informed consent was obtained from all participants.

### 2.2. Immunohistochemistry

All the carcinoma samples were fixed by the addition of 10% neutral buffered formalin, routinely dehydrated, and embedded in paraffin. The immunohistochemical EnVision two-step method and hematoxylin staining were applied. Antibodies included synaptophysin (Syn), chromogranin A (CgA), CD56, phosphoenolpyruvate carboxykinase (PCK), cytokeratin 19 (CK19), cytokeratin 7 (CK7), epithelial membrane antigen (EMA), glypican-3, and hepatocyte.

A number of transcription factors involved in the development of neuroendocrine cells during fetal life can serve as specific histological markers to identify PHNEC. CDX2 is a good marker of midgut origin, TTF1 is expressed in a subset of lung carcinoids, and PDX1 seems to be a good marker of pancreatic origin as well as ISL1. However, these markers can only be used to estimate extrahepatic primary lesions approximately, since they can also be detected in other organizations. Therefore, after the histopathological and immunohistochemical examination, it is still necessary to combine with the comprehensive clinical examination to confirm the diagnosis. The diagnosis of all patients with PHNECs is considered after limiting the possibility of a metastatic focus from an unknown primary NEC.

### 2.3. Observation of Clinical Index

Laboratory data involved alanine aminotransferase (ALT; ≤40 U/L/>40 U/L), aspartate aminotransferase (AST; ≤40 U/L/>40 U/L), hemoglobin (HB; ≤110 G/L/>110 G/L), albumin (≤35 G/L/>35 G/L), alkaline phosphatase (ALP; ≤105 U/L/>105 U/L), *γ*-glutamyl transpeptidase (GGT; ≤50 U/L/>50 U/L), and tumor markers, including *α*-fetoprotein (AFP; ≤400 *μ*g/L/>400 *μ*g/L), carcinoembryonic antigen (CEA; ≤5 *μ*g/mL/>5 *μ*g/mL), carbohydrate antigen 19-9 (CA19-9; ≤35 *μ*g/mL/>35 *μ*g/mL), carbohydrate antigen 125 (CA125; ≤35 *μ*g/mL/>35 *μ*g/mL), and HbsAg (positive/negative). The clinical outcomes included the carcinoma site, carcinoma diameter (≤5 cm/>5 cm), carcinoma location in the liver (right/left), carcinoma number (single/multiple), and treatment (radical operation/others).

### 2.4. Follow-Up Results

Follow-up time ranged between 1 and 60 months (mean = 16.9 months). All patients with PHNECs underwent imaging, histopathology, and immunohistochemical analysis, and long-term follow-up examination, including ultrasound, enhanced chest computed tomography (CT), and upper and lower gastrointestinal endoscopic examination, was conducted. No primary extrahepatic lesions were found. The overall survival time and recurrence-free survival time were defined, respectively, as the interval between the dates of radical operation and death or first recurrence. Data was censored at the last follow-up (June 30, 2017) for patients without death or recurrence.

### 2.5. Statistical Processing

Statistical analysis was performed with the software SPSS® version 23.0 (IBM, Armonk, NY, USA). The data were presented as the median (range) or absolute frequency (%) or the mean ± SD according to the significances of expression. The indexes of immunohistochemical were compared between the primary group and the metastatic group applying the chi-square and Fisher's exact tests. The variance analysis of the effects of the clinical examination, biological investigations, and pathologic indexes on the prognosis was performed by the Kaplan–Meier survival curve and the log-rank test. We used Cox proportional hazard models to assess the significance of the treatment protocol in the multivariate analysis. Values of *p* < 0.05 were considered statistically significant.

## 3. Results

### 3.1. Patients and Clinical Data

Among all reviewed 47 cases, 34 cases belonged to the metastatic group, and 13 cases belonged to the primary group. In the primary group, 8 cases were male patients, and the rest were female patients, corresponding to the ratio of men to women of about 1.60 : 1. The mean age was 53.77 ± 10.55 with a range of 34–76 years. In the metastatic group, 20 cases were male patients, and the rest were female patients, corresponding to the ratio of men to women of about 1.43 : 1. The mean age was 56.79 ± 9.66 with a range of 33–75 years. In the metastatic group, most of the patients (22/34) had symptoms caused by tumor oppression, 10 cases had facial flushing, 8 cases had abdominal pain and diarrhea, and 5 cases had asthma. In the primary group, only 4 cases had abdominal discomfort, 1 case had diarrhea, and all the other cases had no symptoms and were detected incidentally by medical check-ups.

In the primary group, 10 (76.92%) cases had a single nodule, and only 3 (23.08%) cases had multiple nodules. These carcinomas were located in the left lobe of the liver in 3 (23.08%) cases, in the right lobe of the liver in 9 (69.23%) cases, and in both lobes in 1 (7.69%) case. The mean diameter of the carcinoma in the liver was 7.95 ± 3.79 cm with a range of 4–16 cm. The primary carcinoma sites of the metastatic group were mostly the pancreas and stomach. The mean diameter of carcinoma was 4.26 ± 3.05 cm with a range of 1–15 cm. In this group, 25 (73.53%) cases had a single nodule, and 9 (26.47%) cases had multiple nodules in the liver. These nodules were located in the right lobe of the liver in 8 (23.53%) cases, in the left lobe of the liver in 10 (29.41%) cases, and in both lobes in 16 (47.06%) cases (Tables 1–3).

**Figure 1:**
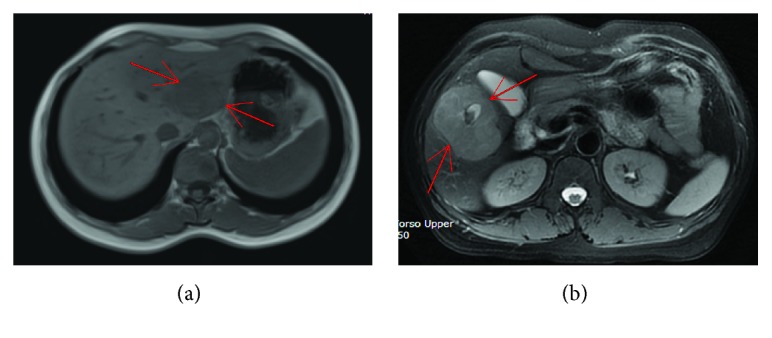
Image examination of primary hepatic neuroendocrine carcinomas. (a) MRI revealed the size of about 5.5 × 4.0 cm, slightly longer T1 and T2 signal masses, a clear boundary, and an uneven internal signal in the left lateral lobe of the liver. (b) Contrast-enhanced MRI revealed an irregular mixed appearance in the right lobe of the liver.

**Figure 2:**
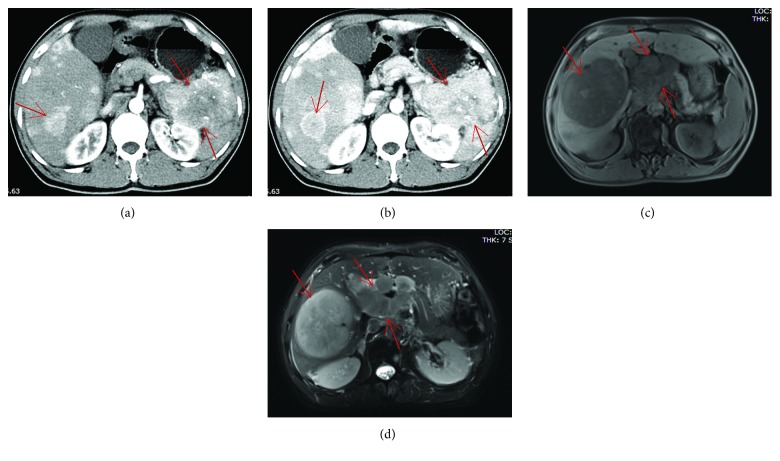
Image examination of gastroenteropancreatic neuroendocrine carcinomas with liver metastasis. (a, b) Contrast-enhanced CT showed the uneven mass of the soft tissue in the pancreas tail with the size of about 8 × 7 cm. The mass was scattered in the point of patchy high-density calcification, and the mass and pancreatic body part of the boundary are unclear. The liver showed multiple sizes of nodules with abnormal enhancement. (c, d) MRI showed multiple round nodules around the head of the pancreas and retroperitoneum, which were similar to the signal and enhancement pattern in the liver, and the pancreatic head was compressed. The liver showed multiple round long T1 signals, a slightly longer T2 signal shadow, and a smooth edge. The larger nodule is located in the right lobe of the liver with a size of about 7.7 × 11.8 × 10.6 cm.

The magnetic resonance imaging (MRI) scan of PHNECs showed slightly longer T1 and T2 signal masses and nodules with clear boundaries. Contrast-enhanced MRI revealed an irregular mixed appearance (Figure 1). The MRI scan of metastatic hepatic NECs showed multiple round nodules in the primary lesions, which were similar to the signal and enhancement pattern in the liver. The contrast-enhanced CT scan of metastatic hepatic NECs showed the uneven mass of the soft tissue in the primary lesions, and the liver showed multiple sizes of nodules with abnormal enhancement (Figure 2).

### 3.2. Immunohistochemistry

**Figure 3:**
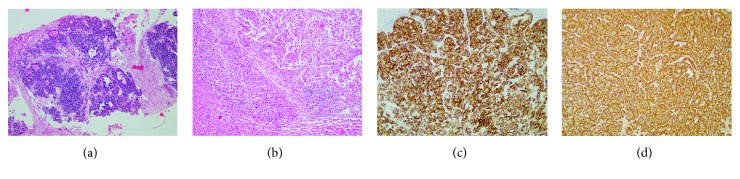
Immunohistochemistry of hepatic neuroendocrine carcinomas. (a) Histological examination showed crowded cells in the liver, striking karyokinesis, and a significantly increased karyoplasmic ratio (×100). (b) Histological examination showed atypical carcinoma cells, partially low regional differentiation, and significantly increased mitoses close to the surrounding liver invasion (×100) caused by the karyoplasmic ratio. (c) Immunohistochemistry revealed that the tumor cells were positive for Syn (×100). (d) Immunohistochemistry revealed that the tumor cells were positive for CgA (×100).

**Figure 4:**
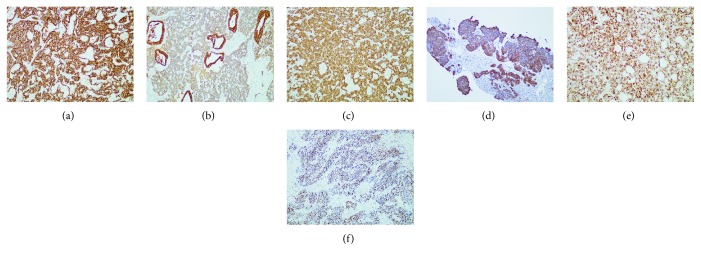
Immunohistochemistry of hepatic neuroendocrine carcinomas. (a) Immunohistochemistry revealed that the tumor cells were positive for CD56 (×100). (b) Immunohistochemistry revealed that the tumor cells were positive for PCK (×100). (c) Immunohistochemistry revealed that the tumor cells were positive for CK19 (×100). (d) Immunohistochemistry revealed that the tumor cells were positive for CK7 (×100). (e) Immunohistochemistry revealed that the tumor cells were positive for EMA (×100). (f) Immunohistochemistry revealed Ki-67 PI of 80% (×100).

In the primary group, Syn, CgA, CD56, PCK, CK19, and EMA showed positive rates of >50% for 100% (12/12), 75% (9/12), 90% (9/10), 87.5% (7/8), 66.67% (6/9), and 80% (4/5), respectively, of the cases. In the metastatic group, Syn, CgA, CD56, PCK, CK7, CK19, and EMA showed positive rates of >50% for 91.18% (31/34), 69.70% (23/33), 76.47% (26/34), 100% (20/20), 68.18% (15/22), 78.26% (18/23), and 78.57% (11/14), respectively, of the cases. No significant difference was determined in either the primary group (*p* > 0.05) or the metastatic group (*p* > 0.05; Table 4, Figures 3 and 4).

### 3.3. Clinical Prognosis Analysis

**Figure 5:**
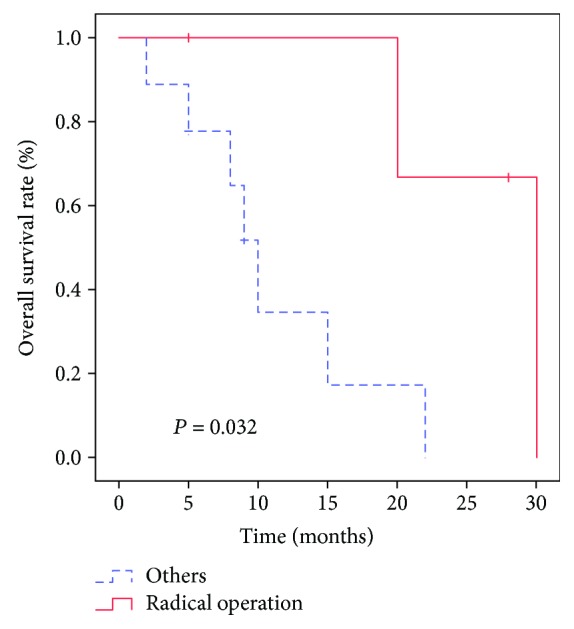
Prognostic values of radical operation in primary hepatic neuroendocrine carcinomas. The survival curve shows that the total survival rate of the radical operation group was higher than that of the other group (*p* < 0.05).

**Figure 6:**
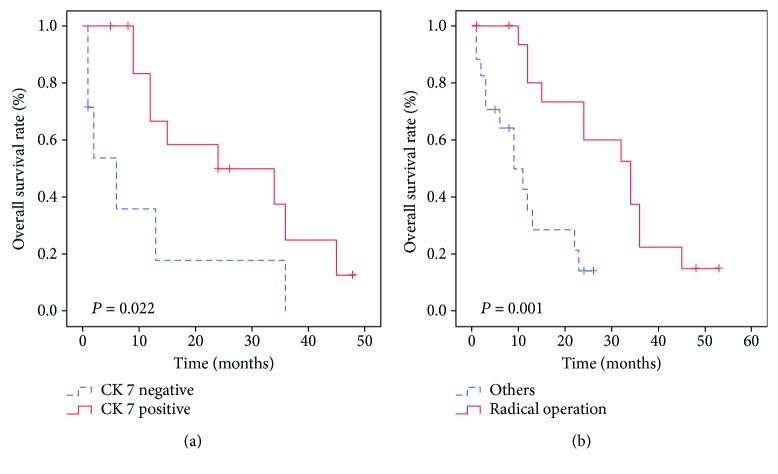
Prognostic values of cytokeratin 7 and radical operation in gastroenteropancreatic neuroendocrine carcinomas with liver metastasis. (a) The survival curve shows that the total survival rate of the cytokeratin 7-positive group is higher than that of the cytokeratin 7-negative group (*p* < 0.05). (b) The survival curve shows that the total survival rate of the radical operation group is higher than that of the other group (*p* < 0.05).

**Figure 7:**
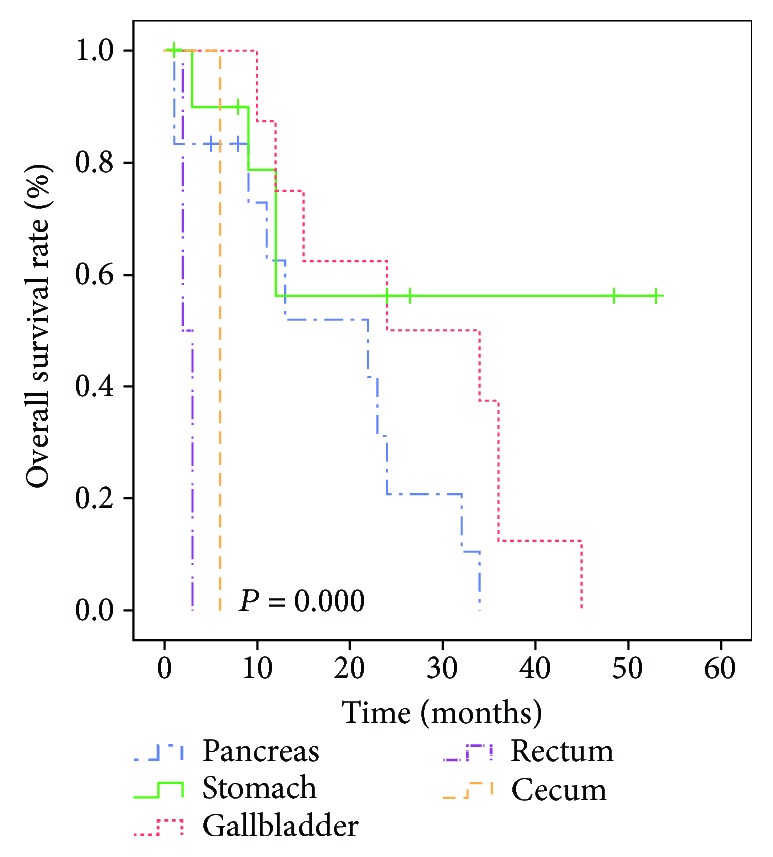
Prognostic values of the primary site of gastroenteropancreatic neuroendocrine carcinomas with liver metastasis. The survival curve shows that the total survival rate of patients with the gallbladder as the primary tumor site is higher than that of the stomach and pancreas, apart from the rectum and cecum (*p* < 0.05).

In the primary group, the mean and median survival times were 12.9 and 9 months, respectively, while in the metastatic group, the mean and median survival times were 18.5 and 12.5 months, respectively. There were two recurrences in the metastatic group with disease-free survival (DFS) times of 23 and 34 months. Univariate analysis showed that the treatment protocol was correlated with the overall survival (OS; *p* < 0.05) in the primary group (Table 5, Figure 5). In the metastatic group, treatment protocol and CK7 were correlated with OS (*p* < 0.05; Table 6, Figure 6). Cox proportional hazard models demonstrated that radical operation was a good independent prognostic factor (*p* < 0.05) for OS (Table 7). We compared the survival of 34 cases of metastatic hepatic NECs from different primary lesions. We found differences in the overall prognosis between them (*p* < 0.05; Figure 7), which may be related to the other metastatic sites of the metastatic group besides the liver foci (Table 8).

## 4. Discussion

GEP NECs with liver metastasis and PHNECs are rare malignancies. Diagnosis of PHNECs is considered challenging in view of the common initial presentation of GEP NECs as metastatic liver lesion. Hepatic neuroendocrine cell may originate from intrahepatic bile duct epithelial cells, heterotopic pancreatic cells, or adrenal tissue [4, 5]. PHNECs can secrete a variety of polypeptides and biogenic amines, including 5-HT, pancreatic polypeptides, gastrin, prostaglandin, and calcitonin. However, only about 5% of patients with the carcinoid syndrome have obvious biological effects. These effects are manifested as skin flushing, asthma, and diarrhea and result from the direct secretion of tumor products, degraded by liver enzymes, into the portal vein circulation, the release of neuroendocrine substances, or the presence of functional defects [6–8]. Clinically, symptoms of epigastric discomfort, loss of appetite, fatigue, and weight loss are often present when the tumor grows to a larger level. No obvious carcinoid syndrome-related symptoms were found in the primary group, whereas the metastatic group was associated with the typical carcinoid syndrome. However, there were many reasons for the carcinoid syndrome in the patients, especially for the metastatic group, because it may involve the corresponding symptoms caused by the metastasis of other parts except the liver or the symptoms of other diseases in the patients.

PHNECs are difficult to diagnose before operation. AFP, CEA, CA19-9, and other tumor markers have no specific diagnostic value in both groups. In this study, all 13 patients in the primary group had normal serum CEA, 1 patient (7.69%) had elevated serum AFP, 2 patients (15.38%) had elevated CA125, and 4 patients (30.77%) had elevated CA19-9. In the metastatic group, the serum AFP levels were normal in all 34 patients, 4 cases (11.76%) had elevated CA125, 9 cases (26.47%) had elevated CEA, and 10 cases (29.41%) had elevated CA19-9. Preoperative diagnosis of PHNECs can only be achieved by the exclusion of extrahepatic primary lesions using imaging. It has been reported that no particular CT/MR imaging feature is specific for PHNECs [9, 10] and the results of PHNEC imaging are often mixed with those of other liver tumors, such as primary hepatocellular carcinoma and primary intrahepatic cholangiocarcinoma [11–13]. Other detection techniques include somatostatin receptor scintigraphy and positron emission tomographic (PET) scanning. For metastatic hepatic GEP NECs, gastroscopy, colonoscopy, endoscopic ultrasound of the pancreas, video capsule endoscopy, and balloon enteroscopy are important examination methods to evaluate for a primary source [14, 15]. Immunohistochemistry has an important value to the diagnosis of NETs. CgA and Syn are generally accepted as highly sensitive immunohistochemical markers for the diagnosis of NETs [16]. It has been reported that Syn is usually positive in NECs, while CgA may be negative [17]. In our study, the positive rate of Syn in both groups was larger than 90%, while the positive rate of CgA was between 60 and 80%. It has been reported that the elevated levels of CgA correlate significantly with carcinoid heart disease, treatment of proton pump inhibitors, chronic atrophic gastritis, and impaired renal function [18, 19]. CK7 is a member of the large CK family and is classified as basal type II [20]. It has been found in most epithelial cells and transitional epithelial cells. Previous studies have shown that CK7 is closely related to tumor prognosis [21]. In our study, the Kaplan–Meier survival curve showed significant differences in CK7 and the prognosis of the metastatic group (*p* < 0.05). The median and mean survival times for a positive expression of CK7 were 15 and 21 months, respectively, and 2 and 8.57 months, respectively, for a negative expression of CK7. These results indicated that positive expression of CK7 is positively correlated with the prognosis of metastatic hepatic GEP NECs, suggesting that CK7 may inhibit tumor growth and that negative or low CK7 expression may predict a poor prognosis of patients, and more attention should be paid to this subset of patients. However, the exact mechanism needs to be studied further.

We found that men were slightly dominant and middle-aged in both groups. However, previous reports also suggested that PHNECs are more common among middle-aged women [22–25]. This discrepancy may be explained by the small number of cases in our study or an increase in the incidence of PHNECs in men. In the primary group, a single nodule was located in the right lobe of the liver, while mostly multiple nodules were located in both lobes of the liver in the metastatic group, which is in accordance with previous reports [26, 27]. Surgical resection is still the first choice for the treatment of PHNECs. Zhang et al. reported that the 5-year survival rate and mean survival time in 58 cases of resected PHNECs were 80% and 148 months, respectively [28], in contrast to 33% and 54 months, respectively, for unresectable neuroendocrine tumors. In our study, four patients with resectable tumors were alive 20.75 months after treatment (range, 5–30 months), while nine patients with carcinomas that could not be surgically removed survived only for 9.44 months (range, 2–22 months), and the 5-year survival rate was 69.23% in the primary group. In the metastatic group, 17 patients with resectable carcinomas were alive 26.47 months after treatment (range, 1–53 months), while other patients with carcinomas that could not be surgically removed survived for only 10.47 months (range, 1–26 months), and the 5-year survival rate was 26.47%. The Kaplan–Meier survival curve also showed that radical surgery was an effective prognostic factor in the two groups. However, the clinical progression of the two groups may vary according to the studied cases. In addition, neuroendocrine tumors are blood-rich tumors and sensitive to ischemia. Therefore, transcatheter arterial chemoembolization (TACE) is also effective in patients who cannot undergo surgery [29, 30]. Local treatment also includes radiofrequency ablation, and chemotherapy is available for patients with distant metastasis. No other protocols for nonradical surgery were summarized and compared to our values in this study. The value of liver transplantation for PHNECs remains a question. Some studies have shown that patients with multiple intrahepatic or extrahepatic metastases or poor liver function can consider transplantation because of the effectiveness of surgery for NECs and the higher survival rate of patients after surgery. Our study exhibits several limitations, including a limited number of patients and a retrospective research design. However, this study still provides valuable findings for the diagnosis and treatment of hepatic NECs in the future.

## 5. Conclusions

Liver NEC is an extremely rare tumor, and no specific clinical features of the disease are reported. Primary hepatic neuroendocrine carcinoma should be considered when no hepatitis or cirrhosis has been diagnosed, AFP is not high, imaging findings suggest solid occupying lesions, liquefaction, and clear boundaries of liver tumors, and other primary lesions have not been found. The final diagnosis depends mainly on pathology and immunohistochemistry, and active surgical treatment is still effective for PHNECs as well as for metastatic hepatic GEPNECs.

## Figures and Tables

**Table 1:** Main demographic, biochemical, and clinical characteristics of the 13 primary hepatic neuroendocrine carcinoma patients.

Variable	Unit	Value
Age	Years	54 (34–76)
Gender	Male	8 (61.54)
ALT	U/L	49 (15–834)
AST	U/L	65 (21–159)
HB	G/L	121 (89–152)
Albumin	G/L	38 (25–49)
ALP	U/L	139 (76–316)
GGT	U/L	90 (15–938)
CA19-9	*μ*g/mL	18 (3–5513.72)
Location in liver	Left	3 (23.08)
PHNEC diameter	cm	5.5 (4–16)
HbsAg	Positive	2 (15.38)
Treatment	Radical operation	4 (30.77)

Data are presented as the median value (range) or absolute frequency (%). PHNECs = primary hepatic neuroendocrine carcinomas, ALT = alanine aminotransferase, AST = aspartate aminotransferase, HB = hemoglobin, ALP = alkaline phosphatase, GGT = *γ*-glutamyl transpeptidase, CA19-9 = carbohydrate antigen 19-9, HbsAg = hepatitis B virus surface antigen.

**Table 2:** Main demographic, biochemical, and clinical characteristics of the 34 gastroenteropancreatic neuroendocrine carcinoma patients with liver metastasis.

Variable	Unit	Value
Age	Years	57.5 (33–75)
Gender	Male	20 (58.82)
ALT	U/L	20.5 (6–6012)
AST	U/L	26.5 (12–4025)
HB	G/L	126.5 (95–156)
Albumin	G/L	38.7 (20.1–47.1)
ALP	U/L	101 (48–634)
GGT	U/L	50.5 (12–1902)
CA19-9	*μ*g/mL	15.3 (1.4–1200)
Location in liver	Left	10 (29.41)
GEP NEC diameter	cm	3.75 (1.2–15)
HbsAg	Positive	5 (14.71)
Treatment	Radical operation	17 (50)

Data are presented as the median value (range) or absolute frequency (%). GEP NECs = gastroenteropancreatic neuroendocrine carcinomas, ALT = alanine aminotransferase, AST = aspartate aminotransferase, HB = hemoglobin, ALP = alkaline phosphatase, GGT = *γ*-glutamyl transpeptidase, CA19-9 = carbohydrate antigen 19-9, HbsAg = hepatitis B virus surface antigen.

**Table 3:** Primary sites of gastroenteropancreatic neuroendocrine carcinomas with liver metastasis.

Primary site	Cases	Percentage
Pancreas	12	35.29%
Stomach	11	32.35%
Gallbladder	8	23.53%
Rectum	2	5.88%
Cecum	1	2.95%

**Table 4:** Immunohistochemistry results of hepatic neuroendocrine carcinomas.

Markers	Group		*p* values^a^
	Primary group	Metastatic group	
Syn			0.557
Positive	12	31	
Negative	0	3	
CgA			1.000
Positive	9	23	
Negative	3	10	
CD56			0.659
Positive	9	26	
Negative	1	8	
Glypican-3			0.175
Positive	4	3	
Negative	5	15	
PCK			0.286
Positive	7	20	
Negative	1	0	
CK7			0.438
Positive	5	15	
Negative	5	7	
CK19			
Positive	6	18	
Negative	3	5	0.654
EMA			1.000
Positive	4	11	
Negative	1	3	

Syn = synaptophysin, CgA = chromogranin A, PCK = phosphoenolpyruvate carboxykinase, CK7 = cytokeratin 7, TTF–1 = thyroid transcription factor, CK19 = cytokeratin 19, EMA = epithelial membrane antigen. ^a^Fisher's exact test.

**Table 5:** Univariate analysis of clinical features in primary hepatic neuroendocrine carcinomas.

Variables	Survival status		*χ* ^2^	*p* values^a^
	Death	Survival		
Gender			0. 081	0.776
Female	3	2		
Male	6	2		
Age (years)			2.212	0.137
≤50	5	1		
>50	4	3		
ALT (U/L)			0.013	0.910
≤40	2	3		
>40	7	1		
AST (U/L)			0.653	0.419
≤40	1	3		
>40	8	1		
HB (G/L)			1.090	0.296
≤110	3	2		
>110	6	2		
Albumin (G/L)			0.141	0.707
≤35	4	2		
>35	5	2		
ALP (U/L)			0.806	0.369
≤105	3	3		
>105	6	1		
GGT (U/L)			0.918	0.338
≤50	2	2		
>50	7	2		
Location			2.518	0.113
Left	0	3		
Right	8	1		
Unknown	1	0		
Number			0.151	0.698
Single	6	4		
Multiple	3	0		
Diameter			0.044	0.835
≤5 (cm)	3	0		
>5 (cm)	4	4		
Unknown	2	0		
CA125 (*μ*g/mL)			0.296	0.586
≤35	7	4		
>35	2	0		
CA19-9 (*μ*g/mL)			0.711	0.399
≤35	7	2		
>35	2	2		
HbsAg			0.412	0.521
Positive	1	1		
Negative	8	3		
Treatment			4.585	0.032^∗^
Operation	2	2		
Others	7	2		

ALT = alanine aminotransferase, AST = aspartate aminotransferase, HB = hemoglobin, ALP = alkaline phosphatase, GGT = *γ*-glutamyl transpeptidase, CA125 = carbohydrate antigen 125, CA19-9 = carbohydrate antigen 19-9, HbsAg = hepatitis B virus surface antigen. ^a^Kaplan–Meier survival curve and log-rank test. ∗ indicates *p* < 0.05.

**Table 6:** Univariate analysis of clinical features in gastroenteropancreatic neuroendocrine carcinoma patients with liver metastasis.

Variables	Survival status		*χ* ^2^	*p* values^a^
	Death	Survival		
Gender			0.027	0.869
Female	11	3		
Male	14	6		
Age (years)			0.708	0.400
≤50	7	2		
>50	18	7		
ALT (U/L)			1.231	0.267
≤40	19	8		
>40	6	1		
AST (U/L)			1.614	0.204
≤40	16	8		
>40	9	1		
HB (G/L)			0.740	0.390
≤110	4	3		
>110	21	6		
Albumin (G/L)			0.235	0.628
≤35	6	1		
>35	19	8		
ALP (U/L)			0.025	0.874
≤105	13	6		
>105	12	3		
GGT (U/L)			0.652	0.419
≤50	10	7		
>50	15	2		
Location in liver			0. 097	0.755
Left	3	7		
Right	6	2		
Unknown	16	0		
Diameter			1.979	0.160
≤5 (cm)	12	5		
>5 (cm)	7	0		
Unknown	6	4		
CA125 (*μ*g/mL)			0.426	0.514
≤35	21	8		
>35	3	1		
Unknown	1	0		
CA19-9 (*μ*g/mL)			0.083	0.773
≤35	16	7		
>35	8	2		
Unknown	1	0		
HbsAg			0.008	0.927
Positive	4	1		
Negative	21	8		
Treatment			10.955	0.001^∗^
Operation	12	5		
Others	13	4		
CK7			5.237	0.022^∗^
Positive	9	6		
Negative	6	1		
Unknown	10	2		

ALT = alanine aminotransferase, AST = aspartate aminotransferase, HB = hemoglobin, ALP = alkaline phosphatase, GGT = *γ*-glutamyl transpeptidase, CA125 = carbohydrate antigen 125, CA19-9 = carbohydrate antigen 19-9, HbsAg = hepatitis B virus surface antigen. ^a^Kaplan–Meier survival curve and log-rank test. ∗ indicates *p* < 0.05.

**Table 7:** Radical operation is an independent predictive factor for gastroenteropancreatic neuroendocrine carcinomas with liver metastasis.

Variable	HR (95% CI)	*p* value^a^
Treatment	0.185 (0.051–0.672)	0.010
Radical operation vs. others		

HR = hazard ratio, 95% CI = 95% confidence interval. ^a^Cox proportional hazard regression.

**Table 8:** Other metastatic sites of the 34 gastroenteropancreatic neuroendocrine carcinoma patients with liver metastasis besides the liver foci.

Primary site	Metastatic sites	Cases
Pancreas	Lung	2
	Bone	2
	Kidney	1
	Omentum	1
	Diaphragm	1

Rectum	Thyroid	1

Stomach	Lung	1
	Bone	1
	Pancreas	1

## Data Availability

We cannot share the data privately because we have not got the publishing license of the data original owner.
